# Rethinking Proteinuria: Resolution of Donor Derived EXT2‐Associated Membranous Nephropathy Following Renal Transplantation

**DOI:** 10.1155/crit/3650246

**Published:** 2026-06-30

**Authors:** Mike Fruscione, Devarsh Desai, Satoru Kudose, Yasir Qazi, Samuel Sultan, Kenneth Lieberman, Michael J. Goldstein

**Affiliations:** ^1^ Division of Organ Transplantation, Hackensack Meridian Hackensack University Medical Center, Hackensack, New Jersey, USA; ^2^ Hackensack University School of Medicine, Nutley, New Jersey, USA; ^3^ Department of Pathology and Cell Biology, Columbia University, New York, USA, columbia.edu; ^4^ Providence St. Joseph Multi-Organ Transplant Center, Orange, California, USA; ^5^ University of Southern California Keck School of Medicine, Los Angeles, California, USA, usc.edu

**Keywords:** membranous nephropathy, proteinuria, renal transplant

## Abstract

Exostosin‐2 (EXT2)‐associated membranous nephropathy (MN) represents a subgroup of immune complex‐mediated renal diseases, where transplantation may facilitate clearance of immune deposits. We present a successful kidney transplantation from a 19‐year‐old deceased donor with previously undiagnosed EXT2‐associated MN into a 75‐year‐old male recipient. Despite significant pretransplant donor proteinuria (> 300 mg/dL) and hypoalbuminemia, renal biopsies at procurement demonstrated well‐preserved renal architecture with minimal interstitial fibrosis. Clinical follow‐up showed improvement in recipient renal function, with serum creatinine decreasing from 3.56 mg/dL preoperatively to 0.96 mg/dL (reference range: 0.7–1.3 mg/dL) within 2 months posttransplant. Proteinuria, initially nephrotic‐range, progressively resolved to subnephrotic by 4 months. At 6 months, the recipient had normalized renal function and sustained resolution of proteinuria. This case demonstrates that removing a kidney from its native pro‐inflammatory environment, coupled with immunosuppression, can facilitate resolution of immune complex‐mediated glomerulopathy.

## 1. Introduction

Membranous nephropathy (MN) is among the leading causes of nephrotic syndrome in adults, accounting for approximately 20%–30% of nephrotic syndrome cases in nondiabetic patients and is a major cause of chronic kidney disease worldwide [1]. MN is characterized, histologically, by immune complex deposition along the glomerular basement membrane resulting in podocyte injury, disruption of the filtration barrier, and subsequent proteinuria. Nephrotic‐range proteinuria in a deceased donor has been regarded as a relative contraindication to renal transplantation due to the risk of graft dysfunction or early graft loss [2].

EXT1/EXT2‐associated MN is connected with systemic autoimmune conditions such as Class V lupus nephritis, with characteristic subepithelial immune complex deposition [3]. Published case reports have described the resolution of immune complex‐mediated glomerulopathies posttransplant due to the removal of the graft from the donor′s pro‐inflammatory and antigenic environment in combination with posttransplant immunosuppressive therapy. [4–6] This case report presents the successful transplantation of a kidney from a deceased donor with previously undiagnosed EXT2‐associated MN.

## 2. Case Presentation

In August 2024, the donor, a 19‐year‐old female, was admitted to a regional medical center in California with progressive left lower extremity swelling and diagnosed with an extensive iliofemoral deep venous thrombosis. The donor′s past medical history included asthma, obesity (body mass index 35 kg/m^2^), and recent pneumonia. The donor was discharged on rivaroxaban and presented 1 week later with worsening respiratory symptoms due to bilateral pulmonary emboli. During the second hospitalization, the donor underwent a thrombectomy, inferior vena cava filter placement, and a heparin infusion.

At admission, the donor′s creatinine was 0.96 mg/dL, peaking at 1.2 mg/dL. Notably, the donor had persistent nephrotic‐range proteinuria (> 300 mg/dL) and hypoalbuminemia (albumin 2.4 g/dL, reference range: 3.5–5.0 g/dL). Serial urinalyses revealed 4+ proteinuria. A hypercoagulable workup was deferred because the patient was actively receiving heparin. The suspected etiology of the thrombosis was the loss of antithrombotic proteins in the setting of nephrotic‐range proteinuria. The donor was treated empirically with 80 mg of prednisone whereas an autoimmune workup for the persistent proteinuria was initiated. During her hospitalization, the donor experienced a seizure, was transferred to the intensive care unit, and was intubated for airway protection. A noncontrast head CT scan revealed diffuse cerebral edema and suspected posterior fossa extra‐axial hemorrhage. The donor lost brainstem reflexes and was declared brain dead.

The donor evaluation was completed by the local organ procurement organization. Based on donor characteristics, the kidney donor profile index was 5%. An abdominal CT scan revealed bilateral striated nephrograms suggestive of acute tubular necrosis. Standard kidney biopsies obtained at the organ procurement showed 40 glomeruli with 0% sclerosis and less than 5% interstitial fibrosis and tubular atrophy.

The recipient was a 75‐year‐old male with Stage V chronic kidney disease secondary to Type II diabetes and hypertension. The recipient had not yet commenced dialysis. The recipient had an estimated posttransplant survival score of 79%, panel reactive antibodies of 0%, and A, B, DR mismatches of 1, 2, 1, respectively. A virtual crossmatch was negative. The right kidney was transported to our center in static cold storage and then placed on a hypothermic perfusion system; final resistance was 0.25 mmHg/mL/min. The transplant was performed in the standard fashion; warm and cold ischemia times were 31 min and 36 h, respectively.

**Figure 1:**
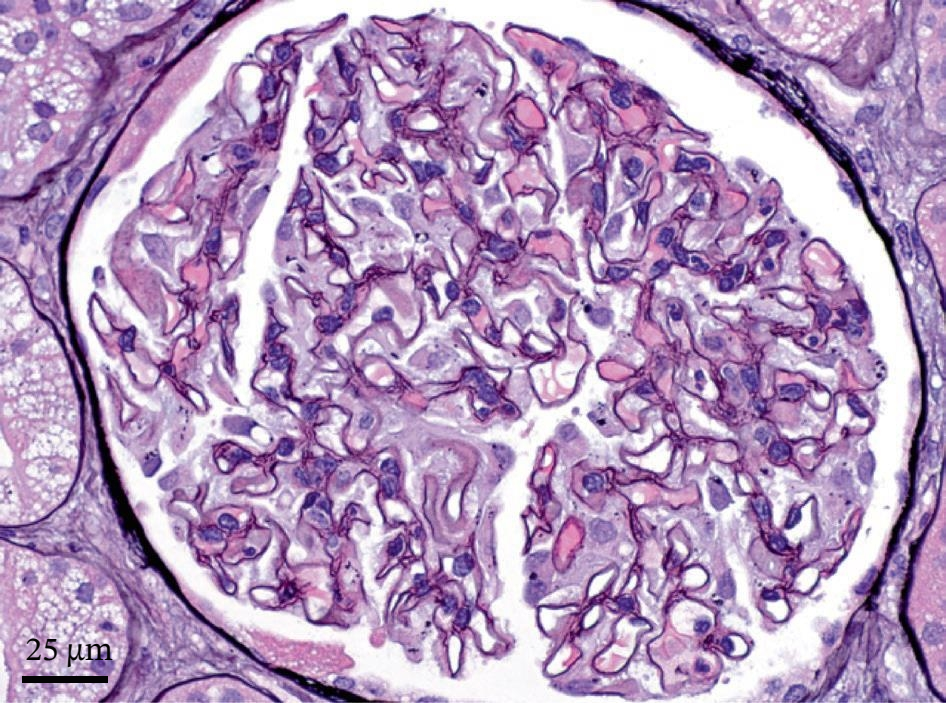
Sampling for light microscopy reveals vacuolization in the glomerular basement membranes (Jones methenamine silver, 600×).

**Figure 2:**
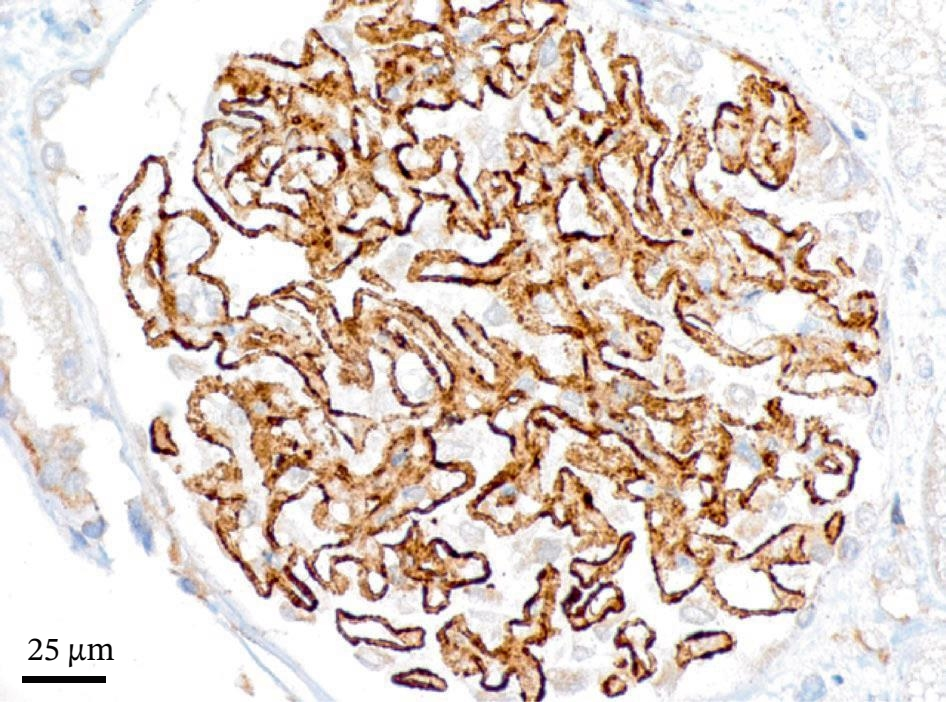
Immunohistochemistry for EXT2 shows strong, diffuse granular staining along the glomerular capillary loops, corresponding to subepithelial immune complex deposits characteristic of membranous nephropathy (600×).

Since the donor work‐up was incomplete and the procurement biopsy lacked diagnostic immunostaining, we performed a biopsy following graft reperfusion. Light microscopy showed diffuse thickening of glomerular capillary loops with Jones methenamine silver stain demonstrating glomerular basement membrane (GBM) vacuolization (Figure 1). Immunofluorescence showed global granular staining for IgG, kappa, and lambda along the glomerular capillary loops. Ultrastructural examination demonstrated diffuse global subepithelial and segmental mesangial deposits. No tubuloreticular inclusions were seen. Immunohistochemistry (IHC) for EXT2 was diffuse and strongly positive in the distribution of subepithelial deposits (Figure 2), whereas stains for THSD7A and NELL1 were negative. These findings confirmed the diagnosis of EXT2‐associated MN.

Immunosuppression included induction with thymoglobulin and methylprednisolone, followed by maintenance with tacrolimus XR and mycophenolic acid.

The recipient′s preoperative creatinine was 3.56 mg/dL (reference range: 0.5–1.1 mg/dL), peaked at 5.79 mg/dL, and improved to 0.96 mg/dL at 2 months posttransplant. Preoperative albumin was 2.7 g/dL, improving to 4.4 g/dL by 4 months. The urine protein–creatinine ratio (UPCR) peaked at 11.8 mg/mg within 1 month and improved to 2.3 mg/mg by 4 months. At 12 months, lab results demonstrated serum creatinine 0.97 mg/dL (reference: 0.7–1.3 mg/dL) and UPCR 1.0 mg/mg (reference: < 0.20 mg/mg). No surveillance biopsies were performed posttransplant; therefore, histological resolution of the disease cannot be definitively confirmed.

**Figure 3:**
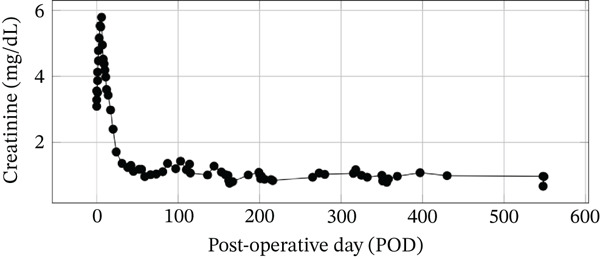
Recipient′s posttransplant serum creatinine.

**Figure 4:**
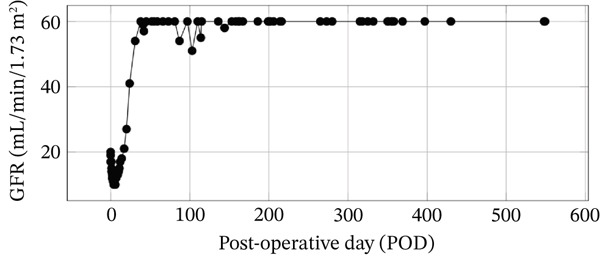
Recipient′s posttransplant estimated glomerular filtration rate.

**Figure 5:**
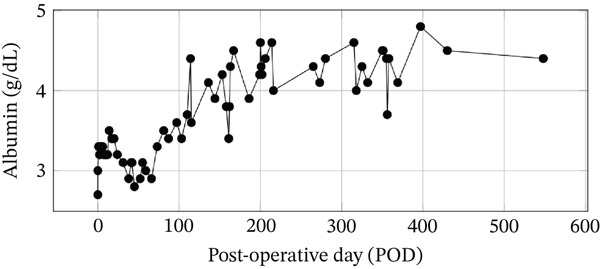
Recipient′s posttransplant albumin.

Figures 3, 4, and 5 shows the trends in recipient serum creatinine and estimated glomerular filtration rate demonstrating rapid normalization of renal allograft function.

**Figure 6:**
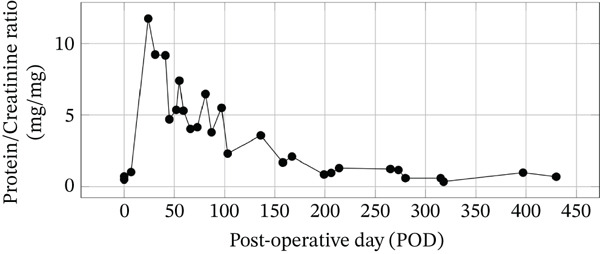
Recipient′s posttransplant urine protein–creatinine ratio.

Figure 6 shows the corresponding progressive resolution of donor‐derived proteinuria, measured via urine protein–creatinine ratio (UPCR), reaching subnephrotic levels by 6 months posttransplant.

At the most recent clinical follow‐up, 18 months posttransplantation, the recipient demonstrated stable allograft function with a serum creatinine of 0.96 mg/dL, eGFR of > 60 mL/min/1.73m^2^, and UPCR of 0.68 mg/mg. Notably, the recipient of the contralateral kidney was a 72‐year‐old female with an initial pretransplant serum creatinine of 5.0 mg/dL, who had an uneventful postoperative course. At 18‐month follow‐up, her serum creatinine was 1.2 mg/dL, eGFR of 52 mL/min/1.73m^2^, and UPCR of 0.21 mg/mg.

## 3. Discussion

Evaluating donor kidneys with underlying glomerular disease poses clinical uncertainty; however, emerging evidence increasingly supports the viability of these grafts. Magoon et al. [4] reported successful transplantation of kidneys with active Class IV lupus nephritis, showing histologic resolution posttransplant. Similarly, recent analyses of transplanted kidneys with preexisting MN report favorable outcomes, including stable graft function and gradual GBM repair [6].

Case reports of renal grafts from donors with MN have also highlighted the microscopic changes associated with recovery. Studies examining histologic timelines demonstrate a progressive reduction in electron‐dense deposits and GBM remodeling, consistent with immune complex clearance and structural repair [5, 6]. In a review of 14 cases of donor‐derived MN, Nuccitelli et al. [6] reported that most recipients maintained stable kidney function with subnephrotic proteinuria at a median follow‐up of 22 months, and suggested persistent proteinuria was likely due to the slow resorption of mesangial deposits. The outcome in our case is consistent with these findings, with normalization of renal function and resolution of proteinuria observed by 6 months posttransplant. Because immune deposit clearance and GBM remodeling can be slow and sometimes incomplete, long‐term monitoring of these allografts remains essential [6].

Despite lacking a comprehensive work‐up of the donor′s underlying proteinuria, the decision to transplant this donor kidney was based on an extensive multidisciplinary discussion between surgeons as well as adult and pediatric nephrologists at our center. The donor′s young age, history, normal creatinine clearance, and unremarkable biopsy were indicative of an acute process causing proteinuria which would likely resolve after transplantation in conjunction with immunosuppression.

The patient′s posttransplant resolution of MN likely reflects both the effects of immunosuppression and the shift to a new immunologic microenvironment. Magoon et al. [4] proposed that removal of the allograft from the donor′s autoimmune milieu and transplantation into a nonautoimmune host facilitates clearance of immune complexes, potentially through macrophage‐ and monocyte‐mediated phagocytosis. The combination of this environmental change and maintenance immunosuppression substantially accelerates the resolution of immune complex‐mediated injury [4, 5]. Together, these processes may restore the glomerular filtration barrier by clearing pathogenic immune deposits directed against EXT2 and reestablishing normal heparan sulfate‐mediated charge selectivity. This interpretation is consistent with prior reports showing that MN can regress once the graft is removed from a setting of circulating autoantibodies. Molina et al. [5] note that pathogenic IgG4 deposits clear relatively early after transplantation into a nonautoimmune host, although the target antigen may persist in the tissue for a longer period. In this context, the granular EXT1/EXT2 staining pattern, which mirrors the distribution of IgG along the GBM, further supports their role as key components of the immune complexes in this MN subtype. The abundance of immune complexes within the GBM, comparable to PLA2R in primary MN, may serve as useful biomarkers of secondary autoimmune‐associated disease [3].

The allograft recovery observed in our case is further supported by the inherently favorable phenotypic profile of EXT‐associated MN. Recent large cohort studies have established that EXT1/EXT2‐positive membranous lupus nephritis represents a distinct clinical phenotype characterized by reduced glomerulosclerosis, interstitial fibrosis, and tubular atrophy when compared to EXT‐negative variants. With the ability to clear immune complex deposits, patients with EXT‐positive disease demonstrate a lower risk of progression to end‐stage kidney disease [7].

## 4. Conclusion

This case highlights the potential of utilizing donor kidneys with underlying immune complex‐mediated glomerular disease. Although we have demonstrated remission of proteinuria in two recipients receiving a renal graft with MN, these results should be interpreted with caution. With histologic and antigenic characterization of renal allografts, donors with MN may offer a means to expand the donor pool. However, further studies are essential to fully define long‐term graft survival and recurrence risk.

**Nomenclature**
EXT2: exostosin‐2
GBM: glomerular basement membrane
MN: membranous nephropathy
UPCR: urine protein–creatinine ratio

## Funding

No funding was received for this manuscript.

## Disclosure

The authors have nothing to report.

## Conflicts of Interest

The authors declare no conflicts of interest.

## Data Availability

The data that support the findings of this study are available on request from the corresponding author. The data are not publicly available due to privacy or ethical restrictions.

## References

[1] Couser W. G. , Primary Membranous Nephropathy, Clinical Journal of the American Society of Nephrology. (2017) 12, no. 6, 983–997, 10.2215/CJN.11761116, 28550082. 28550082 PMC5460716

[2] Pollmann N. S. , Vogel T. , Pongs C. , Katou S. , Morgül H. , Houben P. , Görlich D. , Kneifel F. , Reuter S. , Pollmann L. , Pascher A. , and Becker F. , Donor Proteinuria and Allograft Function in Kidney Transplantation: Short- and Long-Term Results From a Retrospective Cohort Study, Transplant International. (2023) 14, no. 36, 11953, 10.3389/ti.2023.11953. PMC10754218 38156296

[3] Sethi S. , Madden B. J. , Debiec H. , Charlesworth M. C. , Gross L. , Ravindran A. , Hummel A. M. , Specks U. , Fervenza F. C. , and Ronco P. , Exostosin 1/Exostosin 2-Associated Membranous Nephropathy, Journal of the American Society of Nephrology. (2019) 30, no. 6, 1123–1136, 10.1681/ASN.2018080852, 31061139. 31061139 PMC6551791

[4] Magoon S. , Zhou E. , Pullman J. , Greenstein S. , and Glicklich D. , Successful Transplantation of a Donor Kidney With Diffuse Proliferative Lupus Nephritis and Crescents—A Case Report, Nephrology Dialysis Transplantation. (2010) 25, no. 12, 4109–4113, 10.1093/ndt/gfq289. 20817673

[5] Molina Andújar A. , Castrejon de Anta N. , Rodriguez-Espinosa D. , Hermida E. , Larque A. B. , Esforzado N. , Torregrosa J. V. , Cucchiari D. , Blasco M. , Rodríguez-Villar C. , Beck L. H. , García Herrera A. , and Quintana L. F. , Antiphospholipase A2 Receptor Antibody-Positive Membranous Nephropathy in the Kidney Donor: Lessons From a Serendipitous Transplantation, American Journal of Transplantation. (2022) 22, no. 1, 299–303, 10.1111/ajt.16813, 34431212. 34431212

[6] Nuccitelli R. A. , Fernandez H. E. , Husain S. A. , Kudose S. , Batal I. , and Miroslav S. , Donor-Derived Membranous Nephropathy in the Allograft Kidney: A Rare but Probably Underestimated Complication, American Journal of Transplantation. (2024) 24, 2292–2298, 10.1016/j.ajt.2024.08.00. 39127179 PMC12782192

[7] Ravindran A. , Casal Moura M. , Fervenza F. C. , Nasr S. H. , Alexander M. P. , Fidler M. E. , Hernandez L. P. , Zhang P. , Grande J. P. , Cornell L. D. , and Gross L. A. , In Patients With Membranous Lupus Nephritis, Exostosin-Positivity and Exostosin-Negativity Represent Two Different Phenotypes, Journal of the American Society of Nephrology. (2021) 32, no. 3, 695–706, 10.1681/ASN.2020081181, 33478971. 33478971 PMC7920177

